# Medico-Chirurgical Transactions

**Published:** 1817-03

**Authors:** 


*Medico-Chirurgical Transactions, Vol. VII, Part II. Octavo, London, pp. 300. 6 Plates.*

It is in vain for us to deplore the expence of these volumes, which must of *necessity*, especially in these times, exclude them from the perusal of nine-tenths of the profession. £. 1 : 2*s* : 6*d*. for a volume of Transactions — of voluntary contributions, is too much; *Mais n' importe!* Be it our duty, our pleasing labour, to bring their *substance* within the reach of every, the meanest member of medical society.

Seventeen articles are contained in this half volume. We shall analyze them seriatim.

Art. 1. *Observations on the Operation for Artificial Pupil.* By Mr Maunoir, of Geneva, &c. &c. 22 pages.

Our author's introductory letter to Scarpa, will not do for this land of independence! The Marquis of Beaumanoir's eyes will not excite in us a particle more interest than those of the meanest patient that comes to our door for gratuitous advice. If "the most trifling circumstances relative to this case cannot but be highly interesting" to the Medico-Chirurgical Society, it is otherwise with the rigid analysis of the Medico-Chirurgical Journal, where the facts must stand on their own value and interest, unreflected from the brilliant armour of a Marquis, uninfluenced by the lustre of a Professor's name.

The state of this nobleman's eyes in April, 1815, when first seen by Mr. M. was as follows:—They were large, prominent, upper eye-lid rather red and swelled; vessels of the conjunctiva rather turgid; iris presenting, instead of a central pupil, a white spot, the size of a pin's head, formed by the corresponding portion of the crystalline capsule, adherent to the iris of both eyes. It could not be ascertained whether the lens was transparent or opake, as the marquis could only distinguish the transition from darkness to light.

*Operation.* An incision in the lower and external part of the cornea, occupying a full third of the circumference of the membrane. The iris projected a little, and was replaced. Introducing the two blades of his scissors, closed, into the anterior chamber, and then opening them, he caused the pointed blade to penetrate the iris, leaving the blunt blade between that membrane and the cornea; then closing the scissors, a perpendicular incision of the iris resulted, traced on the side of the temple. The formation of the pupil not being complete, the scissors were a second time introduced obliquely, and immediately the pupil appeared of a satisfactory size and form. The second stroke of the scissors had divided the capsula: a small curette was therefore introduced, in order to destroy what adhered of the crystalline to the shrunk and contracted circumference of the old pupil. This attempt did not succeed. Lastly, he effected the passage of a portion of the opake lens, by a slight pressure with a large scoop on the lower part of the globe of the eye. The crystalline, of a cheesy consistence, came out with ease, though not entirely, and left the artificial pupil perfectly black. The old pupil remained closed as before, and was not comprised within the area of the new. A considerable time elapsed before vision was imperfectly restored. Two other cases are related, but not materially differing from this. Professor Scarpa's letter to the author, contains some observations and candid strictures on this operation; but they would not be interesting to one in fifty of our readers.

Art. 2. *Inflammation of the Muscular Structure of the Heart.* By Edward Stanley, Esq. Assistant Surgeon to Bartholomew's Hospital.

This is a very interesting case, and we shall give a perfect analysis of it.

A youth, of Christ's Hospital, ætat. 12, of delicate frame, but hitherto healthy, was suddenly seized, [21st of April,] with febrile symptoms, viz. great bodily heat; quick pulse; white, furred tongue. 22d. Fever much increased; the only pain complained of, was in the left thigh and knee, which ceased before night. In the afternoon, delirium; watchfulness. 23d. Delirium very considerable, sine comate; pupil much dilated, but not insensible to light. Complained of little pain but that in the forehead. In the afternoon, a convulsive fit which soon went off. Evening, omnia exacerbata; no sleep this night. 24th. Much sunk; breathing, first time difficult. Expired at two o'clock in the afternoon. At no period did he complain of pain in any part of the thorax; nor was there any irregularity in the action of the heart or arteries.

Mr. Field, from whose notes this case is taken, states, that as the delirium was similar to what frequently occurs in young persons, there was no indication, until the following morning, which could lead to the supposition of *organic* disease. Effusion was then suspected.

The medical treatment *until that period*, had been directed to counteract general fever, namely, evacuants (query, what evacuants? Was venæsection employed?) and antimonials with the *warm bath*. Leeches were *afterwards* applied to the temples, a blister to the nape of the neck, and several stools procured by a calomel purgative, combined with rhubarb." 325.

Before proceeding to the dissection, we shall make a few remarks on this case. Delicacy towards a respected name will prevent us from saying all we could say; but justice demands candour. 1st. In a young person *with fever and delirium*, shall we be justified in entering into calculations whether the disease be *organic* or functional? If it be *functional*, can we be too active in our measures to prevent its becoming *structural*? If it be *organic*, can we be too active in our measures to prevent *destruction*? It is for Mr. Field to say, whether he bled *usque ad deliquium*, for the relief of the symptoms, on the first appearance of delirium; but whether or not, we will venture to predict that this case will leave an impression on his mind, which will prove a salutary lesson in future. In the second place, we would venture to ask Mr. Field, on what principle the *warm bath* was employed, during "great bodily heat, quick pulse, furred tongue"? This question is not asked by a tyro in the profession. It is asked by one who has witnessed scenes of fever which would astonish Mr. Field. We know the difficulties and embarrasments of practice; but cannot help suspecting that the Brunonian and Cullenian spells still exert a fatal influence over the minds of our metropolitan practitioners. In all acute diseases, venæsection is the *primum mobile* of the cure — all other means are *subordinate* in every sense of the word. "But our effeminate citizens cannot bear the Herculean evacuations of the country." True; but they can bear them till the proper effects are produced. If it requires the abstraction of three pounds of blood to induce syncope in a ploughman, and only twelve ounces in an alderman, the principle is not altered, nor the necessity for inducing syncope done away. But to return.

*Sectio Cadaveris.* Head. Nothing but a general turgidity of the vessels. In the abdomen, every thing healthy. Thorax; lungs sound. In the pericardium, four or five ounces of turbid, serous fluid, with floating flakes of coagulable lymph. The internal surface of the bag covered with a thin reticulated layer of coagulable lymph. Size of the heart natural. The fibres of the parietes exceedingly dark coloured, soft, and loose in their texture. The cut surface, exposed in the section of either ventricle, presented numerous small collections of dark-coloured pus in distinct situations, among the muscular fasciculi. The muscular fibres of the auricles were also softened in their texture, and loaded with blood. Except turgescence of the capillary vessels, no other morbid appearance was visible in the heart.

Mr. Stanley remarks the peculiarity of this case in respect to the anomaly of its symptoms. An inflammation attacks the heart with such violence as to pass immediately into suppuration, and prove fatal in four days; and yet of the symptoms which arose, there was not one directly referable to the affected organ. On the contrary, the attention of the practitioners was diverted from the central organ of the circulation, the actual seat of the disease, to the centre of the nervous system, where there existed no organic derangement. Mr. S. eulogises the sagacity of his friend, Dr. Farre, "who has noticed the reciprocal influence which the sanguiferous and nervous systems have on each other." The influence indeed is great; and Mr. S. might have known that this reciprocity has been beautifully and anatomically demonstrated for many years by Dr. Sanders of Edinburgh, in his annual lectures. We ourselves shall shortly bring into notice some curious instances of this principle; and shew the necessity of attending to the state of the *vascular* system in various derangements of the *nervous* system, where the *former* is little suspected of exerting an influence.

Art. 3. *Wounded Peroneal Artery.* By G. J. Guthrie, Esq.

This vessel is so well protected against all the common accidents of domestic life, that any disease of it will seldom require an operation. But in military life every part is exposed; and it is probable that wounds of the peroneal artery have not unfrequently led to amputation of the limb.

CASE. H. V. of the German legion, received a musket ball in the battle of Waterloo, which entered the right leg immediately behind and below the inner head of the tibia, inclining downwards, and under, or before a part of the soleus and gastrocnemius muscles, coming out through them, four inches below the head of the fibula, towards the side of the calf of the leg. Little inflammation, and no immediate hæmorrnage followed. On the 1st of July, a considerable hæmorrhage took place, and was suppressed by the tourniquet. In the morning of the 2d, it returned, and an operation was determined on. The limb, from the frequent applications of the tourniquet, was swelled; coagulated blood was forced under the soleus, and florid blood issued from both openings.

*Operation.* An incision, seven inches in length, in the axis of the limb, taking the shot-hole as a central point, and carried by successive strokes through the gastrocnemius and soleus muscles towards the peroneal artery. The inflammation, extravasation, and incipient sphacelus of the parts, rendered it impossible to discover the vessel, though arterial blood issued from the parts. A transverse incision was then made outwards from the shot-hole to the edge of the fibula. Mr. G. could now pass a tenaculum under the spot whence the blood issued, but still the wounded artery could not be distinctly seen, nor separated. A small needle therefore, armed with two threads, was passed at a sufficient distance from the tenaculum, and one end drawn, when the hæmorrhage ceased. Another ligature was applied below, and the operation finished in the usual manner. The man recovered.

ART. 4. *Gun-Shot Wound, and Fracture of the Tibia, wherein a Seton promoted the Cure.* By JOHN BOGGIE, Esq. Surgeon to the Forces.

CASE. An officer of the 95th, received a musket shot through the right leg in the battle of Orthes. The ball entered on the anterior spine of the tibia, two inches and a half from its articulation with the foot, passing obliquely outwards through the substance of the tibia, injuring the fibula, and coming out towards the exterior edge of that bone. The healing process went on during a month, when he fell down, and fractured both bones at the site of the wound. Violent inflammation, high fever, and delirium followed, and were reduced by large evacuations, poultices, fomentations, &c. All went on well again, for a time, when the process of consolidation was completely stopped, attended with swelling of the limb, and thin, watery discharge. As there was no appearance of reunion of the bones, a seton was passed through the canal formed in the bone by the ball. A favourable change took place almost immediately; many small portions of bone came away, and in about five weeks the seton was withdrawn, and soon after both wounds healed.

Art. 5. *New Method of Operating for the Cure of External Aneurism, &c. &c.* By Philip Crampton, Esq. Surgeon General to the Army in Ireland.

This is a very important communication, and shall receive a proportionate minuteness of analysis.

The experiments, first of Petit, Pouteau, and Kirkland —lately of Jones, on the arteries of brutes, have attracted the attention of all surgeons. The latter gentleman, it is well known, drew the conclusion, " that if the ligature does not completely cut through the middle and internal coats all around the artery, adhesion cannot take place, &c." *Treatise,* p. 70. But our author justly remarks here, that this conclusion rests on no other foundation, than experiments on the arteries of quadrupeds; *in all of which,* the internal and middle coats were ruptured by the ligature. Where then is the *negative* evidence? Besides, conclusions drawn from the analogy supposed to subsist between the arteries of man and quadrupeds, are fallacious. 1st. Because the adhesive process is more quickly and certainly executed in all parts of quadrupeds than in man. 2dly. Wounds of arteries in the *former* are so prone to unite, that aneurisms *cannot* be induced thereby. 3dly. The arteries of quadrupeds are not liable to that peculiar change of structure which predisposes to aneurism, and renders the operation so uncertain in man. Hence, then, although the process of reparation is the same in both species of animals, there is a *difference* of result; namely, that in animals it is *always* successful — in man too often otherwise.

Our author next proceeds to shew, from various observations and experiments, 1st. That the obliteration of an artery can very certainly be effected, independently of the rupture or division of any of its coats. 2nd. That this operation of the ligature is not essential to the process, and sometimes defeats it.

In proof of these Mr. C. brings forward the instances on record of arteries obliterated by the pressure of tumours, as the subclavian and carotid. In Mr. Freer's experiments, the pressure of a tourniquet for four days obliterated the radial artery in horses. Mr. Hunter observed that the mere exposure of the tibial artery of a dog to the air for an hour, excited such inflammation as completely obstructed the canal.

*Exp.* 1. The carotid of a sheep was compressed by a flat tape, the ligature being drawn no tighter than to bring the internal surfaces of the artery in contact, and removed in twenty-six hours. Twenty-five hours after this, the animal was killed. For an inch and a half above and below the ligature, the parietes of the artery were thickened by coagulated lymph between its coats: beyond the ligature, the artery was much reduced in size.

"The canal was so completely obstructed at the place of the ligature as to be impervious to water."

On slitting open the artery, a band of coagulated lymph, slightly tinged with blood, was found adhering to the internal coat, and occupying its whole circumference.

*Exp.* 2. In this instance, the carotid of a sheep was compressed so gently by a slip of tin, that the current of blood was not entirely arrested. At the expiration of sixty hours, the parts were examined. The artery, for the extent of three inches, was bedded in coagulated lymph. When the slip of tin was removed, the vessel retained the flattened form at the place where it had been compressed. At this point the internal surfaces were in contact, but did not cohere. The area of the tube seemed to be obliterated by a thickening of the coats, or more correctly speaking, by an effusion of coagulated lymph between the cellular and middle coat. There was a large coagulum in the lower portion of the artery.

Mr. C. thinks that the division of the inner and middle coats not unfrequently *prevents* the obliteration of the artery, for the following reasons:—Ulceration or rupture of these coats, whether with or without a morbid dilatation of the tube itself, is the immediate cause of aneurism; consequently, when we operate, a similar violence is inflicted on an artery in a condition the most favourable for the production of the disease. But we shall not follow Mr. C. through all his reasonings: we shall proceed directly to facts.

Case 1, October 1814. Francis M'Donnel was received into the Military Infirmary, near Dublin. For three months previously, he had complained of pain in the calf of his leg, which, on the 20th of September, incapacitated him for duty. He was then seized with the most excruciating pain in the ham, which, on examination, was found to be swelled, and throbbing violently. His pulse was now upwards of 120, remarkably full and strong. He had not slept for many nights, and was occasionally delirious. The aneurism was of an unusually large size, distending the ham, forcing up the calf of the leg, and forming a large pulsating tumour under the fascia of the vastus internus muscle. Forty ounces of blood were taken from his arm, his bowels were freed, and an evaporating wash was applied to the tumour.

*Operation,* 14*th of October.* The femoral artery was laid bare at the usual place, by an incision about three inches in length, and a tape one eighth of an inch in breadth was passed under it by means of an aneurism needle. The ends of the ligature were passed through the holes in the foot of the "*presse artere,*" and then crossed through the hole in its stalk. The artery was gently compressed, till the pulsation ceased in the tumour. The ligature was then secured, and the lips of the wound approximated by adhesive plaster. The operation took up twelve minutes. In about twenty minutes the man complained of excruciating pain in the calf of his leg. He took thirty-five drops of laudanum. At half-past two he still complained. The ligature was relaxed as far as it pleased. No pulsation returned; the pain abated. At six in the evening, he vomited freely.

15th. Had considerable pain during the night; but it ceased at one, A. M. and he slept two hours: pulse 96; has had four stools. Says the limb felt cold until two in the morning, when it became warm and comfortable. Relaxed the ligature completely; no pulsation in the ham. The instrument and ligature were entirely withdrawn on the 17th, and he joined his regiment in two months after the operation. He died suddenly shortly afterwards of aneurism of the aorta.

CASE 2. Mr. Smith, 26 years of age, had hitherto been healthy. In August 1814, he perceived a small pulsating tumour in the ham. In December, it began to increase rapidly, and the pain became intense. In January, 1815, he took to his bed, and Dr. Dease visited him in the beginning of February, when he found him greatly emaciated; his pulse 126, full, and bounding; said he felt as if his whole body was shaken by the strong pulsations of his arteries. External pressure had been tried by Mr. Adrian, but the pulsation in the ham could not be stopped.

*Operation by Dr. Dease, 27th of February,* 1815.—The artery was laid bare in the usual place, and the "presse artere" was applied, as in the case of M'Donnell. The patient was not sensible of any peculiar sensation when the artery was compressed by the ligature. In five hours and a half the ligature was completely loosened. In about a minute, a deep and obscure pulsation was discerned in the tumour; it became more distinct every moment. The ligature was immediately tightened and the pulsation ceased. In twenty-four hours after the operation, and nineteen since the circulation had been last interrupted, the ligature was completely loosened. No pulsation returned, and he rapidly recovered.

Since the preceding, a case of great interest occurred in Dublin.

On Friday the 23d of February, 1816, the femoral artery was tied (in the manner recommended by Mr. Travers) for an aneurism of the posterior tibial artery.

On Monday the 26th, the case appeared to be doing well, all pulsation having ceased in the tumour; and the temperature of the limb being natural, a slight attempt was made to loose the knot, which proving ineffectual, was not persevered in.

On Wednesday the 28th, at eight o'clock, A. M. the ligature appearing to lie loosely in the wound, was withdrawn without resistance, the loop remaining perfect; a violent hæmorrhage came on at one P. M. the same day.

The artery was secured again about three inches higher up. No alteration in the temperature of the limb followed. On Friday night, the patient became delirious, and died on the following morning.

On dissection, the artery was found to be completely divided at the place of the first ligature: its extremities had retracted to the extent of three quarters of an inch. The mouth of the upper extremity of the tube was circular, its area was not contracted, nor were its coats thickened; but it was imperfectly obstructed by a coagulum about half an inch long, which seemed to adhere partially to the internal coats. There was no appearance of lymph having been effused from the cut edge of the divided coats. The lower extremity of the tube presented similar appearances, with this difference, that it was more perfectly obstructed by a coagulum.

Here then the division of the internal and middle coats had been effected by the application of the ligature; and yet the complete division of the tube, in consequence of the sloughing or ulceration of the external coat, on the morning of the fifth day. After the division of the tube, hæmorrhage was only delayed by the presence of a slightly adherent coagulum.

Time alone must decide on the merits of Mr. Crampton's proposal. At the time of writing this, (November 1816) we have only heard of two instances in this metropolis, where the operation was performed according to Mr. Travers's plan. They were both ultimately successful; but secondary hæmorrhage took place, and three days were required for compression on the artery. Our readers know that Dr. Hutchinson's case was unsuccessful. *Vide* No. 12, p. 464.

Art. 6.—*A Sketch of the Medical History of the* 1*st Battalion of the* 1*st Regiment of Foot Guards, during the Winter of* 1812--13. By John Bacot, Esq.

There is not in this long paper of fourteen pages, a single line worthy of commemoration for the private practitioner. Peace to its manes!

Art. 7.—*Microscopic Observations on the Structure of Bone.* By John Howship, Esq.

We regret that the nature of the subject, and the reference to plates, totally prevent our giving any account of this very interesting paper. As a physiological, rather than a practical subject, it is less adapted for transfusion into the pages of our Journal; but not less capable of heightening the estimation in which the talents of Mr. Howship have long been held by us, and the medical world. The plates, as usual, are exquisite.

A short paper on the Condition of the Bones in Rickets; by Edward Stanley, Esq. follows, and is illustrated by three well executed plates. This is a subject of *curiosity*, and involves no practical question whatever. We shall, therefore, refer the *curious* to the volume itself.

Art.

Art. 9.—*Further Observations on Contractions succeeding to Ulceration of the Skin.* By Henry Earle, Esq.

Sixteen pages are here occupied in stating what might easily have been conveyed in sixteen lines. The first case was contraction of the fingers from a burn; the cicatrix was removed from one finger at a time, and considerable extension of the fingers was the result. The second case was a retraction of the thumb from the same cause; removal of the cicatrix was very beneficial. The third case was Mr. Brodie's; a boy who had a bridle or fold of skin on the anterior part of the neck, from the healing of a sore formed by an extensive burn. The bridle was in a longitudinal direction, drawing down the lower lip, cheeks, and angles of the mouth, keeping the chin much depressed, preventing the jaws being closed, and occasioning considerable deformity. The cicatrix, which was transverse and broad, with the condensed cellular membrane under it, was removed by an operation. The edges of the wound were drawn together by adhesive plaster, and a broad collar of pasteboard was applied round the neck over the dressings. In fine, he was able to close the jaws, and scarcely any deformity remained. This case of Mr. Brodie's occupies little more than three quarters of a page, and forms a singular contrast with the wire wove ductility and tenuity of Mr. Earle's. This gentleman says that the case is doubly interesting, " as it is the first in which the *cicatrix* was removed for a contraction of the integuments of the neck." It may be so; but the removal of a *contraction* itself, by dividing the cicatrix, is fresh in our memories, though we cannot call to mind the periodical publication in which the case is related, and a plate given of the parts anterior and subsequent to the operation. We acknowledge, however, that Mr. Earle has established a very useful fact in surgery; and for which he deserves our thanks, but he should study *brevity*.

Art. 10.—*Hernia of the Dura Mater, complicated with Hydrocephalus.* By the same.

W. ——, an infant child, was born with a transparent globular tumour at the back of the head; which, in eight days, attained the size of a small billiard ball. It bore a resemblance to spina bifida, and appeared to consist of an expansion of dura mater, containing serum, in consequence of deficient bony matter, or other support at this part. The head was not large or malformed. The pupils obeyed the impression of light; there was no strabismus; no loss of power in the limbs, nor any one symptom of pressure on the brain, except constipation of the bowels. The tumour was punctured with a common needle, when ʒiij of limpid serum oozed out. The tumour soon regained its size, was again squeezed, and ʒiij came away. Thus it continued to fill and be emptied, sometimes with the needle, sometimes the lancet, and sometimes a fine trocar, from the 11th February till the 23d of March, when ulceration of the sac, derangement of the bowels, foreran death, which took place unaccompanied by convulsions, or any one decided symptom of inflammation or effusion on the brain.

Dissection shewed a slight effusion of bloody serum to some extent round the opening in the bone. The tun. arach. appeared rather opake, and raised by subjacent fluid contained in the cellular texture of the pia mater. The membranes and substance of the brain were particularly void of red vessels. The medullary structure was remarkably soft. The lateral ventricles were enlarged, and contained four ounces of water. The sac communicated with the ventricles.

"Although this case terminated unfavourably, it proves that the radical cure may be attempted *with impunity* in similar cases."

We think this doubtful; but do not object to the attempt, both here and in spina bifida.

Art. 11.—*Extra Uterine Fœtus in the Fallopian Tube.* By G. Langstaff, Esq.

A woman, previously enjoying good health, was seized at two o'clock in the morning with a violent pain in the lower part of the abdomen, accompanied by sickness at the stomach and disposition to faint. Some brandy and water was exhibited, and produced trifling mitigation of the symptoms. At five o'clock they were aggravated. Her pulse became rapid, small, and intermitting; countenance ghastly; a cold clammy sweat pervaded the whole body; the intellectual functions declined. She sunk rapidly, and died in seven hours from the attack.

*Dissection.* On opening the abdomen, arterial-looking blood, chiefly fluid, covered the intestines, and occupied the pelvic region. The quantity measured two quarts. When sponged out, the viscera were found natural but pale. The right Fallopian tube was dilated to the size of a hen's egg, and had burst in two places. The distention of the tube had commenced two inches from the fimbriated extremity. The internal surface retained its plicated arrangement, while the portion of tube, from the enlargement to the angle of the uterus was obliterated. The Fallopian contents were next examined. A chorion and amnios were discovered, with a fœtus of about eight weeks, floating in the liq. amnii. The right ovarium contained a gelatinous-looking mass. There were several corpora lutea in the left ovarium, one of which shewed the vesicle removed from thence in the conception previous to the last. The uterus was considerably larger than in the unimpregnated state. There was a quantity of gelatinous matter in its cavity and neck. The internal surface was very vascular. The rest of the body was completely free from organic disease.

The most remarkable circumstance here is the want of decidua in the uterus. The case is curious, but void of any practical utility.

Art. 12. *Observations on Tetanus.* By Dr. Dickson, Physician to the Fleet.

Dr. Dickson's object in this paper is to excite greater attention to particular symptoms, especially to the prevention of "that torpid state of the bowels, which so *undeviatingly* precedes the attack." We think this a very unqualified expression. We have witnessed no trifling number of tetanic affections, and are quite certain that very many of them, consequent on wounds and injuries, come on so suddenly, that preceding torpor of the bowels could have nothing to do with the complaint. We refer Dr. Dickson to Dr. Hutchinson's late work, where the only case of locked jaw there related, occurred in four or five hours after the wound, the man being in previous good health. Vide *Med. Ch. Journal*, No. xii. page 461.

The examples that came within our author's knowledge were six or seven soldiers under the care of navy surgeons; and these occurred a few days after the attack of the 8th of January, 1815, at New Orleans. The men had been exposed to the cold night-air and heavy rains. The danger was in proportion to the rapidity and violence of the attack, and the disease terminated fatally within the second, third, or fourth day. Dr. D. is able to detail the particulars of only one case, "from having been favoured with some observations by Mr. Boyd, the surgeon of the hospital ship;" and this was an African, who had lost a great part of his foot by sloughing, after a frost-bite. We hope we need not, at this time of day, detail over again the symptoms of tetanus, which are to be found in every system of medicine. It is sufficient to say, that poor Blacky died, as hundreds had done before. The beginning of the case is no where *detailed;* and all we learn is, that he died "the next morning!" During the progress of the complaint, the patient took above three ounces of laudanum. Of this, however, there is some doubt, as Mr. Boyd and the assistant disagree in their statements; but Dr. D. gives the preference to the testimony of the latter. The medicine was commenced in doses of 100 drops, and was gradually augmented every two hours. ℥xiv of rum made into punch were also given, but no degree of intoxication could be induced. He had formerly lost a leg in the service; and from this and other circumstances, Dr. D. is inclined to think, that a greater susceptibility of impressions, and consequently a predisposition to tetanic affections, exists in those who have been previously wounded. This is not an unreasonable supposition. The pulse, as is usual, was not much affected till the ulterior stages of the disease.* "The torpor of the intestines which precedes and accompanies this disease, is highly deserving of attention." Our author thinks, that "the late infrequency of locked jaw in the West Indies is chiefly owing to the greater freedom with which purgatives have been employed of late years." We are much surprised, that a gentleman of Dr. Dickson's talents should indulge in so very unfeasible an hypothesis. How does he account for the infrequency of yellow fever itself? and how does he account for the dreadful ravages of that disease in the summer and autumn of 1816, during a period of profound peace, and when few or no raw and unseasoned Europeans were arriving there? Alas! more effective reasons than a few doses of calomel and jalap must be sought for, before we unravel these mysteries.

In the early stages of traumatic tetanus, Dr. D. places great reliance on venæsection and free purging. But we cannot see on what principle Dr. Dickson combined rum punch with these remedies. Dr. Dickson is not an empiric; he acts always on fixed principles. If he considers rum punch as efficient in curing the disease, surely he must allow that it ought, on the same principle, to be useful as a *preventive;* and if it were a preventive, few tetanic affections would be seen in the West Indies!

* Is it not remarkable that no dissection is given?—Ed.

The pathology of tetanus is acknowledged to be involved in obscurity. M. Larrey states, that he found the pharynx and œsophagus much contracted, and their internal membranes red, inflamed, and covered with a reddish viscid mucus. Dr. M'Arthur, as will presently be shewn, found the intestines much inflamed; and in two instances, a yellow waxy fluid, of a peculiar offensive smell, covered their internal surface.

### *Dr. M'Arthur's Cases.*

Case 1. An African boy, ætat. 14, (at Barbadoes) had suddenly been taken ill of cold and sore throat. To these succeeded dysphagia, and inability to open wide the jaws; but no spasms at this time. Six grains of calomel and ten of ex. colocynth to be taken, and an acid gargle to be used. The cathartic not operating in a few hours, he was ordered some sulphate of magnesia, when both trismus and opisthotonos were developed, and became more aggravated by every attempt to swallow. To take eight grains of calomel every two hours, with mercurial frictions to the jaw, neck, arms, and thighs. The cold affusion. Within the first twenty-four hours he took 70 grains of calomel, and rubbed in two ounces of the ointment. The cold affusion had been applied eight times, and several enemas had been thrown up, and all retained. The spasms increasing, the warm bath was substituted for the cold affusion. The mercurial frictions were continued, and he took 60 drops of laudanum every two hours, in as much wine as he could swallow. A blister was applied to the surface of the abdomen. He died in extreme misery.

*Dissection.* The small and large intestines were much inflamed, and in many places approaching to a state of gangrene. Other abdominal viscera sound.

It was somewhat curious, that, although Dr. M'Arthur repeatedly pressed the abdomen with his hand during the absence of spasm, no pain was complained of.

Case 2. Jer. Collins, ætat. 30, was admitted into Barbadoes Hospital on the 1st of June. The jaws were not completely locked; painful contractions of the muscles of the neck and back; skin perspirable, bowels bound. The first phalanges of the fore and middle fingers had been amputated about twelve days before. The tetanic symptoms appeared on the 31st of May. Laudanum in large doses had been given on board, with frictions and the warm bath. In the hospital, glysters, warm baths; eight grains of calomel every two hours.—*June* 2. Unable to swallow the smallest quantity of fluid. Soon after an opiate glyster was thrown up he died, about sixty hours from the attack.

*Dissection.* Strong marks of inflammation on the internal and external coats of the stomach. Villous coat covered with a peculiarly offensive yellow matter. Small intestines much inflamed. Caput coli and colon bordering on a state of sphacelus. Convex surface of the liver redder than usual; concave surface livid. Gall-bladder turgid with bile. Œsophagus inflamed to within two inches of the pharynx; its mucus yellow, resembling that found in the stomach. Pharynx and parts about the neck and jaw of natural appearance. Lungs healthy.

CASE 3. W. Merrit, ætat. 30, admitted 25th of September, 1807, with an extensive sloughing ulcer on the ankle and back of the foot. After various changes and proposals, he at length consented to amputation, when exhaustion rendered success doubtful. 20th November, the limb was amputated below the knee. Dec. 3, prelusive appearances of tetanus. 4th, considerable rigidity of the muscles of the jaw, and spasms of the muscles of the neck and back. Countenance mournful. Venæsection ad ℥xviij; the warm bath; a dose of pulv. jal. comp. 5th. The warm bath last night was followed by profuse sweating for six hours. No stool; no alteration in the symptoms. To take four grains of calomel every three hours, till the bowels are opened. 6th. The spasms increased. The pulse rose after a venæsection of ℥xvj. Had a stool yesterday after an enema, and *one spontaneous stool* this morning. Skin perspirable; appearance of the stump unfavourable. Calomel, antimonial powder, and camphor, thrice a day. Warm bath night and morning. 7th, little alteration. 8th and 9th, ditto. 10th, passed a bad night; spasms severe; a gangrenous spot on the anterior edge of the stump. *One free stool.* Medicines to be repeated; and to take ʒj tinct. opii at bed-time. 11th, died at two o'clock.

*Dissection.* Stomach much drawn up under the diaphragm. Intestines in several places inflamed, chiefly the ileum: stomach and intestines inflated with air. No mark of disease in the œsophagus, pharynx, or larynx.

CASE 4. William Harris was badly wounded on the 4th of December, 1807. The arm was removed at the shoulder joint on the 5th. The under flap gangrened and sloughed. Dec. 16th, he was restless and somewhat delirious; pulse sunk; granulations of the wound pale; *bowels open*. Wine and porter to be continued. An anodyne at night. 17th. Symptoms of locked jaw. Took 60 drops of tinct. opii at bed-time, and repeated it in the night. Spasms rapidly increased. Warm bath last night. Died at nine o'clock this morning.

*Dissection*. Stomach singularly contracted; internal coat exhibiting appearances of inflammation, and much yellow-coloured mucus adhering to it. Portions of the intestines inflamed, particularly the ileum. A very extraordinary yellow fluid, resembling that in the stomach, was in great abundance in every part of the internal canal, which, upon cutting into the intestine, effervesced on coming in contact with the external air. Liver diseased. Thorax and head not examined.

We have detailed these four cases, in preference to Dr. Dickson's, because the dissections are appended to them; the only circumstance that can now give value to cases of tetanus.

We appeal to our readers, whether they do not militate against Dr. Dickson's assumption respecting the *undeviating* torpor of the bowels. They certainly go far to prove, however, that inflammation very frequently attends or supervenes on the complaint. We ourselves have long entertained an opinion, that tetanus, hydrophobia, and that species of cholera termed *mort de chien*, have a strong affinity. We are convinced that Mr. Tymon's case, which made so much noise, was *mort de chien*, and not hydrophobia.

Dr. M'Arthur observes, that the inflammation in these cases was different from that in enteritis or the endemial fevers of the country. In enteritis, the intestines often adhere to one another by layers of coagulable lymph, recently thrown out; curdled matter and pus are also sometimes found. In the tetanic inflammation there are no adhesions, no formation of pus. In the endemial fever, the whole of the small intestines are more uniformly inflamed; the internal coats of the stomach and intestines exhibiting gangrenous spots and patches; the colon always contracted, but very rarely inflamed. In tetanus, the colon was equally inflamed with the small intestines, and not contracted. The yellow matter found in the stomach and intestines is very remarkable. "Is it to be considered a feature of the disease? or was it the consequence of the laudanum taken?"

We hope Dr. M'Arthur will shake off his repugnance to writing. Few men have richer stores of knowledge locked up. Let him remember the parable of the "Talents!"

ART. 13.—Curious *Imperfection of Vision*. By W. NICHOLL, M. D. [four pages.]

The articles of *curiosity* are not for our market. We shall not offer many of them to our readers. The subject of the present case is a boy eleven years of age. His eyes are grey, with a yellow tinge surrounding the pupil.

"He does not call any colour green. Dark bottle green he calls brown. Light yellow he calls yellow, but darker yellows and light browns he confounds with red. Dark brown he confounds with black. Pale green he calls light red. Common green he terms red. Light red and pink he calls light blue. Blue, both dark and light, he calls blue." *Sic omnia ad finem!*

> "Black spirits and white,
> Blue spirits and grey."

( To be concluded in our next. )

